# Prevalence and mortality rate of healthcare-associated infections among COVID-19 patients: a retrospective cohort community-based approach

**DOI:** 10.3389/fpubh.2023.1235636

**Published:** 2023-08-10

**Authors:** Soha Fakhreddine, Mirna Fawaz, Salwa Hassanein, Alissar Al Khatib

**Affiliations:** ^1^Department of Infectious Diseases, Saint-Georges Hospital, Hadat, Lebanon; ^2^Department of Nursing, Faculty of Health Sciences, Beirut Arab University, Beirut, Lebanon; ^3^Department of Nursing, Faculty of Health Sciences, Almoosa College, Al Ahsa, Saudi Arabia; ^4^Department of Community Health Nursing, Cairo University, Cairo, Egypt

**Keywords:** COVID-19, severity, HAI, prevalence, mortality rate

## Abstract

**Background:**

The prevalence of HAI among COVID-19 patients ranged between 4.8% and 42.8% with the highest occurrence observed in critically ill patients. The present study aimed to evaluate the clinical features of HAI in severe and critical COVID-19 patients, their microbiological characteristics, and the attributable risk factors.

**Methods:**

This is an analytical observational, retrospective single-center, cohort study that included 723 patients with severe-critical COVID-19 admitted to Saint George Hospital between September 2020 and February 2021. Data collection included demographic variables (sex, age), comorbidities, laboratory findings, HAI types and agents, COVID-19 treatment modalities, hospitalization settings, length of stay, and mortality rate. Data was analyzed using SPSS version 25.

**Results:**

The prevalence of patients developing HAI was 7.3% (53 of 723). Five types of nosocomial bacterial infections were tracked noting ventilator-associated pneumonia (41.26%), catheter-associated urinary tract infection (28.6%), hospital-acquired pneumonia (17.44%), catheter-related bloodstream infection (6.35%), and bloodstream infection (6.35%). Binary logistic analysis showed that HAI are statistically affected by four factors noting patients' age (*p* = 0.039), Length of Stay (*p* < 0.001), BIPAP (*p* = 0.019), and mechanical ventilation (*p* < 0.001). The risk of having HAI increases 3.930 times in case of mechanical ventilation, 2.366 times in case of BIPAP, 1.148 times when the LOS increases 1 day, and 1.029 times when the age is higher with 1 year.

**Conclusion:**

Since the prevalence of HAI is high among severe and critical COVID-19 patients, it is important to prepare a treatment with diagnostic, preventative, and control measures for this infection.

## 1. Introduction

According to the World Health Organization (WHO) there have been 767,518,723 Coronavirus Disease 2019 (COVID-19) confirmed cases and 6,947,192 death cases worldwide since December 2019 and up to June 29, 2023 (1). Healthcare-associated infections HAI are infection(s) obtained after a hospital stay for at least 48 h, that should not be presented during the time of admission. According to the Centers for Disease Control and Prevention (CDC) and the National Healthcare Safety Network (NHSN), HAI can be classified into several categories; some infections are related to devices like catheter-associated urinary tract infection (CAUTI), catheter-related bloodstream infection (CRBSI) and ventilator-associated pneumonia (VAP). Others are related to procedures like surgical site infections (SSI) or antimicrobial use like clostridium difficile infection (CDI) and multidrug-resistant organism (MDRO) infections (2).

During intensive care unit stay, bacterial, and fungal superinfection has been described in other outbreaks of severe acute respiratory syndrome (SARS), but there is slight data available regarding COVID-19 patients (3). Many writers acknowledge the significance of HAI, but conclusive data is still required. The reported prevalence of HAI ranges between 4.8% and 42.8% with the highest occurrence observed in critically ill patients (4). The most common infections were respiratory, bacteremia, and urinary tract infections, and the most common germs in respiratory infections were Gram-negative bacteria (50.00%), followed by Gram-positive bacteria (26.92%), viruses (11.54%), fungi (7.69%), and others (3.85%) (5). A retrospective study conducted in China describing intensive care unit (ICU) patients with COVID-19 has shown that carbapenem-resistant *Enterobacterale* (CRE) and MDRO were the most bacteria found among HAI. Patients more than 60 years of age and those intubated for more than 13 days have a higher risk of HAI among COVID-19 patients (6). Concerning the Middle East and Lebanon, to our knowledge, there are insufficient studies on the prevalence of HAI among COVID-19 patients. Hence, the present study was conducted to assess the prevalence of HAI among severe and critical COVID-19 patients admitted to a hospital in Lebanon.

## 2. Aim of the study

The present study aimed to evaluate the clinical features of HAI in severe and critical COVID-19 patients, their microbiological characteristics, and the attributable risk factors.

## 3. Materials and methods

### 3.1. Research design and setting

This is an analytical observational, retrospective single-center, cohort study including 723 patients with severe-critical COVID-19 admitted to Saint Georges Hospital to both ordinary wards and ICU between September 2020 and February 2021.

### 3.2. Sample size

Due to a lack of studies concerning HAI among COVID-19 patients in Lebanon and the Middle East, the prevalence used here to determine the expected sample size was extracted from a study done in a tertiary hospital in China and it was 12.5% (7).

The sample was calculated by using the equation: n = [z^2^ × p × (1-p)]/d^**2**^

Where:

n = sample size.
z = critical value at 95% confidence level which is 1.96.
p = anticipated prevalence which is 12.5% (0.125).
d = degree of precision set at 5% (0.05).
n = [1.96^2^ × 0.125 × (1–0.125)]/0.05^2^ = 168.

Our sample size was large compared to the calculated one. Seven hundred and twenty-three patients, hospitalized in Saint Georges Hospital-Hadat, between September 1, 2020, and February 28, 2021, who were classified as severe and critical cases and who had positive severe acute respiratory syndrome coronavirus-2 polymerase chain reaction (SARS-CoV-2 PCR) test through nasal swab or respiratory secretions were included in this study.

### 3.3. Inclusion criteria

1. Adults: Patients more than 18 years old with positive SARS-CoV-2 PCR and who are classified as severe and critical cases.
2. Severe illness: patients who have respiratory rate >30 breaths per minute, or saturation < 94 on room air, or ratio of arterial partial pressure of oxygen to fraction of inspired oxygen < 300 mmHg, or lung infiltrates >50% (8).
3. Critical illness: patients who have respiratory failure, septic shock, and/or multiple organ dysfunction (8).

### 3.4. Exclusion criteria

1. Patients less than 18 years.
2. Patients transferred from another hospital or discharged to another hospital.
3. Patients admitted to the hospital with positive serology tests but negative PCR.
4. Patients who are classified as mild and moderate cases.

### 3.5. Data collection

Data collection was done through medical files and electronic records of all patients admitted to Saint Georges Hospital between September 2020 and February 2021: demographic characteristics, medical comorbidities, smoking habit, type of HAI, type of bacteria and their resistance pattern, COVID-19 treatment used, and clinical outcome.

HAI included in the study were documented by the presence of a positive culture of sputum, deep trachea aspirate, blood, and urine samples with clinical indication of infection. The infectious diseases team was consulted for all COVID-19 patients including those with suspected infections and positive cultures. Cultures of clinical samples were ordered by physicians and infectious diseases specialists when they suspected the presence of HAI. Then infections were classified and defined according to CDC (2).

### 3.6. Data collection tools

The data collection form was designed to collect data using an electronically validated database (Microsoft Excel). The data collection form was valid and reliable and included the following variables:

- Demographic variables (sex, age).
- Smoking habit and comorbidities: Diabetes Mellitus (DM), coronary artery disease (CAD), Hypertension (HTN), Atrial fibrillation (Afib), Heart Failure (HF), Asthma, chronic obstructive pulmonary disease (COPD), Chronic Kidney disease (CKD), Liver disease, Hematologic malignancy, Solid malignancy, Immunosuppressive therapy.
- Type of HAI: catheter-related bloodstream infection (CRBSI), bloodstream infection (BSI), catheter-associated urinary tract infection (CAUTI), Hospital-acquired pneumonia (HAP), and ventilator-associated pneumonia (VAP).
- Type of bacteria: Gram-positive (*Staphylococcus aureus, Enterococcus faecalis*) vs. Gram-negative (*Acinetobacter baumanii, Pseudomonas aeruginosa, Stenotrophomonas maltophilia*, and *Enterobacterales* like *Escherichia coli, Klebsiella pneumoniae, Proteus mirabilis*, and *Citrobacter koseri*.
- Type of resistance: Extended Spectrum Beta lactamase (ESBL), Methicillin-resistant *S. aureus* (MRSA), CRE and Multidrug Resistant (MDR) *A. baumanii*. Note that *A. baumanii* is considered MDR when it demonstrates resistance to at least one agent in 3 or more classes of antibiotics (9).
- Laboratory results (collected at hospital admission): White blood cell (WBC), polymorphonuclear neutrophils (PMN) cells, Lymphocytes (lymph), C-reactive protein (CRP), Procalcitonin (PCT), D-Dimer, Ferritin, interleukin- 6 (IL6).
- COVID-19 treatment: Baricitinib, Steroids, convalescent plasma transfusion, and Remdesivir.
- Oxygen support: nasal cannula (NC), face mask (FM), non-rebreather face mask (NRFM), high flow nasal cannula (HFNC), bilevel positive airway pressure (BIPAP), non-invasive ventilation (NIV), mechanical ventilation (MV).
- Hospitalization setting: Ordinary ward vs. ICU and Length of stay (LOS) (days of hospital stay starting from admission date till discharge or death date).
- Outcome: Clinical improvement vs. mortality.

### 3.7. Ethical consideration

The Institutional review board (IRB) at Al-Rassoul Hospital approved the study. The identity of the patients remains anonymous, and the research data remains confidential.

### 3.8. Data analysis

SPSS 25.0 was used to examine the dataset (IBM Corp, Armonk, NY, USA). The dependent variable was “HAI”. As a first step, a descriptive analysis was enrolled to assess the prevalence of HAI, their types, and the causal agents for the infection. Results were presented as frequency and proportions.

Bivariate analysis was enrolled to assess the factors affecting the HAI. All the secondary variables were presented in the function of HAI. Tests used in the bivariate analysis were the Chi-square test, Fisher exact test, Student *t*-test, and Mann-Whitney test. Finally, a binary logistic regression model was used to predict factors affecting HAI. A statistically significant association was set at 5% (*p* < 0.05 was considered as significant).

## 4. Results

**Figure 1:**
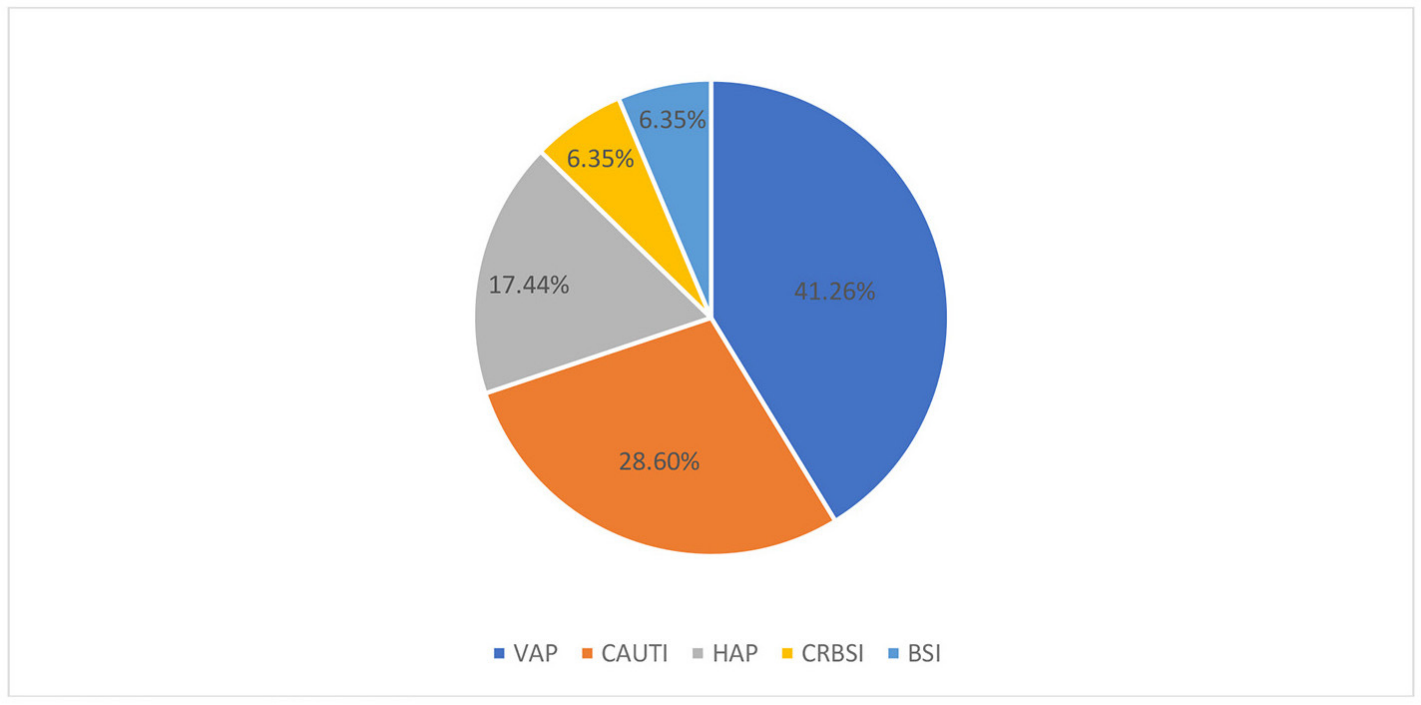
Healthcare-associated infections types in COVID-19 patients. HAI, Healthcare-associated infections; VAP, Ventilator-associated pneumonia; CAUTI, Catheter-associated urinary tract infection; HAP, Hospital acquired pneumonia; CRBSI, Catheter-related bloodstream infection; BSI, Bloodstream infection.

**Figure 2:**
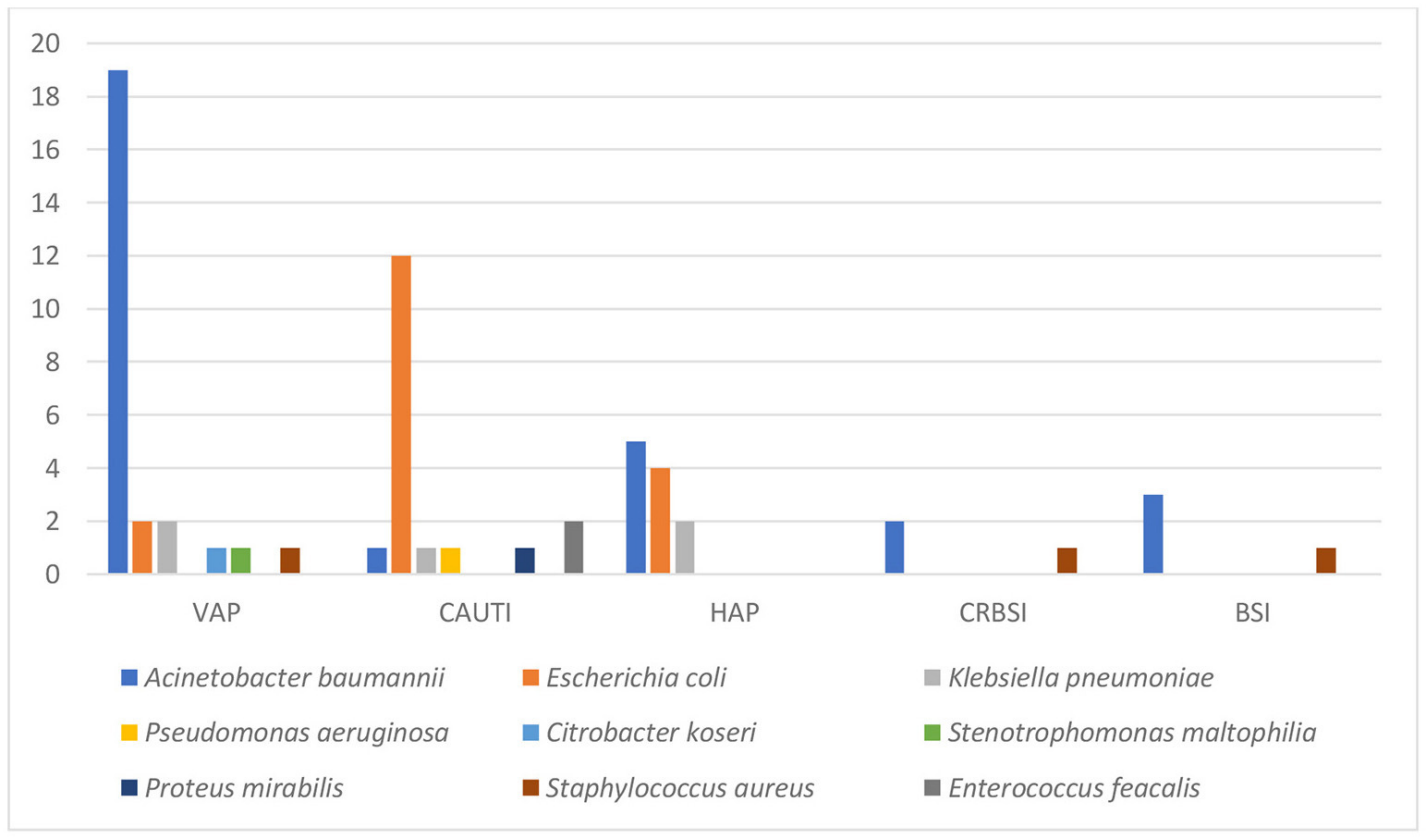
Healthcare-associated infections types and the causative agents. HAI, Healthcare-associated infections; VAP, Ventilator-associated pneumonia; CAUTI, Catheter-associated urinary tract infection; HAP, Hospital acquired pneumonia; CRBSI, Catheter-related bloodstream infection; BSI, Bloodstream infection.

Seven hundred twenty-three severe-critical COVID-19 patients admitted to Saint Georges Hospital between September 2020 and February 2021 were included in this study. The prevalence of patients with at least one HAI was 7.3% (53 out of 723 patients developed HAI). The total number of HAI was 63 with 8.7% prevalence. Five types of HAI were tracked noting VAP (26 cases−41.26%), CAUTI (18 cases−28.6%), HAP (11 cases−17.44%), CRBSI (4 cases−6.35%), and BSI (4 cases−6.35%; Figure 1). All HAP infections were caused by Gram-negative bacteria (11 cases−100%). The top two infectious agents were *Acinetobacter baumannii* (5 cases−45.5%) and *E. coli* (4 cases−36.4%). The majority of VAP infections were caused by Gram-negative bacteria (25 cases−96.2%). The top two infectious agents were *A. baumannii* (19 cases−73.1%) and *E. coli* (2 cases−7.7%). CRBSI were caused by Gram-negative bacteria (2 cases−50%) which were *A. baumannii* and by Gram-positive bacteria (2 cases−50%) which were *S. aureus*. BSI were caused by Gram-negative bacteria (3 cases−75%) noting *A. baumannii*, and by Gram-positive bacteria (1 case−25%) noting *S. aureus*. The majority of CAUTI infections were caused by Gram-negative bacteria (16 cases −88.9%). The top infectious agents were *E. coli* (12 cases−66.7%), *K. pneumoniae* (1 case−5.6%), *P. aeruginosa* (1 case−5.6%), and others (Figure 2).

**Figure 3:**
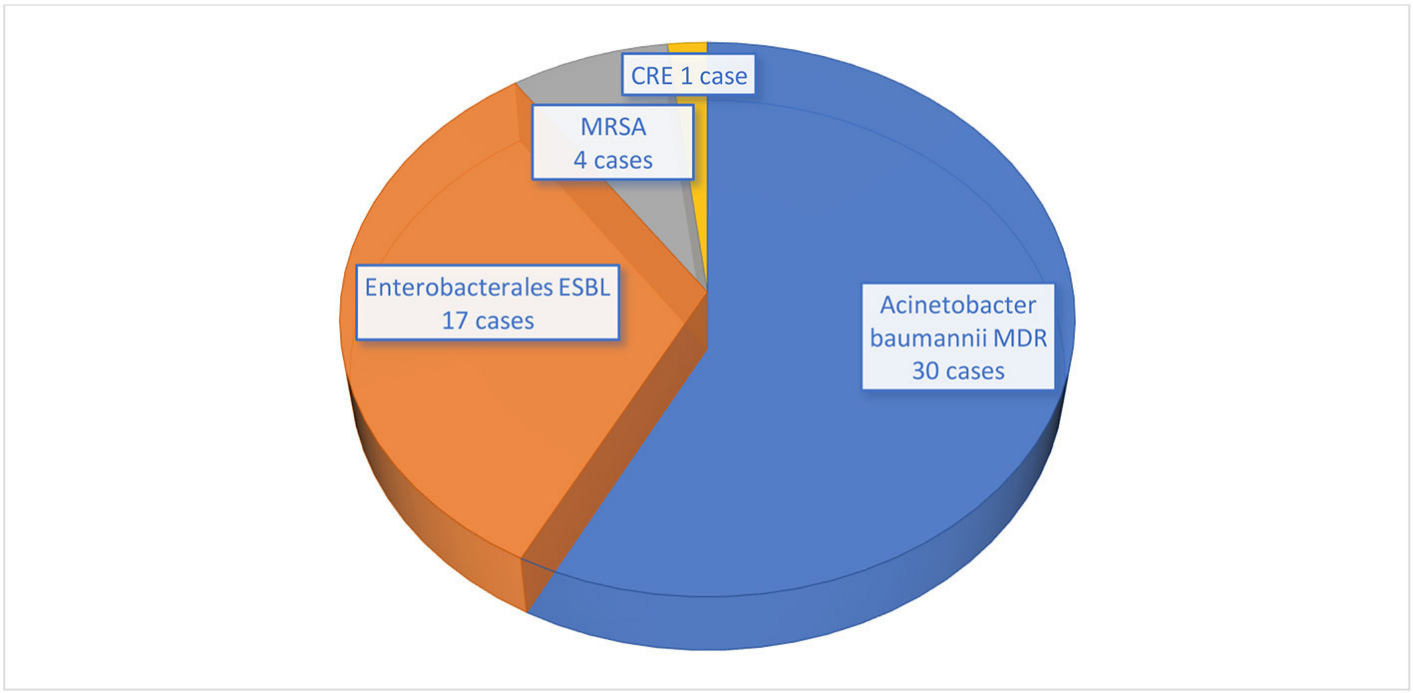
Antimicrobial resistance profile of microorganisms isolated from COVID-19 patients. MDR, Multidrug resistant; ESBL, Extended spectrum beta-lactamase; MRSA, Methicillin-resistant *Staphylococcus aureus*; CRE, Carbapenem-resistant *Enterobacterales*.

Figure 3 shows the microorganisms with their resistance type. All *A. baumanii* (30 cases) were MDR. Among 25 *Enterobacterale* bacteria, 17 were ESBL and 1 was CRE. All *S. aureus* (4 cases) were MRSA (Figure 3).

Table 1 shows the comparison between patients with and without HAI. Patients with HAI were distributed between 35 (66%) males and 18 (34%) females, with no statistically significant difference between HAI and patients' sex (*p* = 0.676). On the other hand, there was a statistically significant difference among age groups (*p* = 0.001). Patients with HAI were older (average age = 69 ± 10.9) than patients not showing HAI (average age = 61.4 ± 16.1; Table 1). HAI were significantly associated with DM (*p* = 0.001), HTN (*p* = 0.003), CAD (*p* = 0.032), and CKD (*p* = 0.007). Moreover, HAI were higher in patients with CKD (11.3%), CAD (35.8%), DM (52.8%), and HTN (71.7%; Table 1).

**Table 1:** Comparison of COVID-19 patients with and without HAI.

**Variables**	**HAI (*N* = 53)**	**Without HAI (*N* = 670)**	***P*-value**
**Demographic characteristics**			
Age, years, median (IQR)	70 (60–76.5)	62 (50–74)	**0.001** ^b^
Male sex, *n* (%)	35 (66)	461 (68.8)	0.676^a^
Female sex, *n* (%)	18 (34)	209 (31.2)	0.676^a^
**Comorbidities and smoking habit**, ***n*** **(%)**			
DM	28 (52.8)	203 (30.3)	**0.001** ^a^
HTN	38 (71.7)	336 (50.1)	**0.003** ^a^
CAD	19 (35.8)	153 (22.8)	**0.032** ^a^
Afib	0 (0)	41 (6.1)	0.064^c^
HF	6 (11.3)	42 (6.3)	0.155^a^
Asthma	1 (1.9)	23 (3.4)	1.000^c^
COPD	3 (5.7)	27 (4)	0.477^c^
CKD	6 (11.3)	24 (3.6)	**0.007** ^a^
Liver disease	1 (1.9)	1 (0.1)	0.141^c^
Immunosuppressive therapy	2 (3.8)	31 (4.6)	1.000^c^
Solid malignancy	1 (1.9)	21 (3.1)	1.000^c^
Hematologic malignancies	1 (1.9)	11 (1.6)	0.602^c^
Smoking	15 (28.3)	148 (22.1)	0.297^a^
**Laboratory data at admission date, median (IQR)**			
WBC × 10^3^/μL	21.77 (15.28–33.36)	11.04 (8–16.21)	**< 0.001** ^d^
PMN %	93.5 (88–95.65)	85.1 (77.63–90.68)	**< 0.001** ^d^
Lymph %	3.2 (1.65–7.8)	10.05 (5.73–16.98)	**< 0.001** ^d^
CRP mg/dl	23.25 (17.35–29.9)	14.5 (8.6–21.7)	**< 0.001** ^d^
PCT ng/ml	0.85 (0.29–2.09)	0.19 (0.08–0.55)	**< 0.001** ^d^
IL-6 pg/ml	65.6 (15.7–138.9)	42.56 (13.38–96.35)	0.594^d^
D-Dimer ng/ml	4,343 (1416.5–7621.7)	1119.5 (599.03–2,602)	**< 0.001** ^d^
Ferritin ng/ml	1368.5 (734.75–2295.75)	855 (432.75–1907.95)	**0.019** ^d^
**Hospitalization settings**			
Ordinary wards, *n* (%)	37 (69.8)	590 (88.1)	**< 0.001** ^a^
ICU, *n* (%)	40 (75.5)	162 (24.2)	**< 0.001** ^a^
ICU stay duration, days, median (IQR)	13.5 (8.25–21.75)	6 (3–11)	**< 0.001** ^d^
LOS, days, median (IQR)	20 (14–25.5)	7 (5–11)	**< 0.001** ^d^
**COVID-19 treatment**, ***n*** **(%)**			
Baricitinib	16 (30.2)	109 (16.3)	**0.010** ^a^
Remdesivir	41 (77.4)	466 (69.6)	0.232^a^
Plasma transfusion	11 (20.8)	108 (16.1)	0.381^a^
Decadron (steroids)	53 (100)	660 (98.5)	1.000^c^
**Oxygen support**			
NC, *n* (%)	6 (11.3)	299 (44.6)	**< 0.001** ^a^
FM, *n* (%)	5 (9.4)	122 (18.2)	0.133^c^
NRFM, *n* (%)	40 (75.5)	220 (32.8)	**< 0.001** ^a^
HFNC, *n* (%)	9 (17)	40 (16)	**0.002** ^a^
BIPAP, *n* (%)	30 (56.6)	77 (11.5)	**0.001** ^a^
NIV, *n* (%)	5 (9.4)	8 (1.2)	**0.001** ^a^
MV, *n* (%)	37 (69.8)	117 (17.5)	**< 0.001** ^a^
Duration of MV, days, median (IQR)	9 (4–13)	3 (1–7.25)	**< 0.001** ^d^
Mortality rate, *n* (%)	38 (71.7)	5135 (20.1)	**< 0.001** ^a^

Tests done using chi-square test (a), Student t-test (b), Fisher-exact test (c) and Mann-Whitney test (d). Bold: statistically significance set at 5%.

Patients with HAI had a higher WBC (median [IQR] = 21.77 × 10^3^/μL [15.28–33.36]), a higher PMN (median [IQR] = 93.50% [88.00–95.65]), had a higher CRP (median [IQR] = 23.25 mg/dl [17.35–29.90]), a higher D-dimer (median [IQR] = 4343.00 ng/ml [1416.50–7621.70]), a higher Ferritin (median [IQR] = 1368.50 ng/ml [734.75–2295.75]), a higher PCT ng/ml (median [IQR] = 0.85 [0.29–2.09]) comparing with patients with no HAI with significant difference of *p* < 0.001. Patients with HAI had a lower Lymph (median [IQR] = 3.20% [1.65–7.80]) comparing patients with no nosocomial bacterial infections (median [IQR] = 10.05 [5.73–16.98]) (*p* < 0.001; Table 1). Among all patients who developed HAI, 75.5% were in the ICU compared to 24.2% of patients with no HAI (*p* < 0.001). ICU stay duration was too much higher in patients with HAI (median stay = 13.5 days) compared to patients with no HAI (median stay = 6 days; *p* < 0.001). In addition, LOS in the hospital was too much higher in patients with HAI (median stay = 20 days) compared to patients with no HAI (median stay = 7 days; *p* < 0.001; Table 1). As shown in Table 1, patients who developed HAI were more exposed to Baricitinib (30.2%) compared to patients with no HAI (16.3%; *p* = 0.010). There was no statistically significant difference between HAI and the treatment with Remdesivir (*p* = 0.232), Plasma transfusion (*p* = 0.381), and Decadron (*p* = 1.000; Table 1). Patients with no HAI were given oxygen supplementation via nasal cannula (44.6%) more than patients with HAI (11.3%) (p < 0.001). Patients with HAI needed more NRFM (75.5%), HFNC (17%) and BIPAP (56.6%), and NIV (9.4%), compared to patients with no HAI (*p* < 0.005). Moreover, patients with HAI needed more mechanical ventilation (69.8%) compared to patients with no HAI (17.5%; *p* < 0.001). In addition, the time of mechanical ventilation was higher in patients with HAI (median duration = 9 days) compared to patients with no HAI (median duration = 3 days; *p* < 0.001; Table 1). The mortality rate was much higher among patients who developed HAI (71.7%) compared to patients with no HAI (*p* < 0.001; Table 1). Finally, a binary logistic analysis was enrolled to evaluate the factors affecting the HAI. The model included all the variables which were statistically associated with the HAI in the bivariate settings. Results were figured in Table 2 and the adjusted model is shown in Table 3 with the odds ratio (OR) and the 95% of confidence interval (CI). HAI are statistically affected by four factors and the risk of having HAI increases 3.930 (95% CI [1.875–8.237] times in case of MV (*p* < 0.001), 2.366 (95% CI [1.149–4.871] times in case of BIPAP (*p* = 0.019), 1.148 (95% CI [1.103–1.194] times when the LOS increases 1 point (1 day) (*p* < 0.001), and 1.029 (95% CI [1.001–1.056] times when the age is higher with 1 point (1 year; *p* = 0.039).

**Table 2:** Binary logistic analysis for the factors affecting HAI.

	**B**	**S.E**.	**Wald**	**Df**	**Sig**.	**OR**
Age	0.019	0.016	1.445	1	0.229	1.019
DM	0.467	0.365	1.635	1	0.201	1.595
HTN	0.296	0.448	0.436	1	0.509	1.344
CAD	−0.028	0.411	0.005	1	0.945	0.972
ICU	−0.460	0.627	0.538	1	0.463	0.631
LOS	0.145	0.023	40.212	1	0.000	1.157
Baricitinib	−0.137	0.458	0.089	1	0.765	0.872
NRFM	0.184	0.441	0.175	1	0.676	1.202
HFNC	−0.126	0.518	0.059	1	0.808	0.882
BIPAP	0.907	0.425	4.557	1	0.033	2.477
MV	1.610	0.566	8.095	1	0.004	5.005
Constant	−6.740	1.170	33.164	1	0.000	0.001

Dependent variable: HAI.

Variable(s) entered in the model: Age, DM, HTN, CAD, ICU admission, LOS, Baricitinib, NRFM, HFNC, BIPAP, MV.

**Table 3:** Adjusted binary logistic analysis for the factors affecting HAI.

	**B**	**S.E**.	**Wald**	**Df**	**Sig**.	**OR**	**95% C.I. for EXP (B)**	
							**Lower**	**Upper**
Age	0.028	0.014	4.250	1	0.039	1.029	1.001	1.056
LOS	0.138	0.020	45.593	1	0.000	1.148	1.103	1.194
BIPAP	0.861	0.368	5.464	1	0.019	2.366	1.149	4.871
MV	1.369	0.378	13.141	1	0.000	3.930	1.875	8.237
Constant	−7.165	1.056	46.034	1	0.000	0.001		

Dependent variable: HAI (No/Yes).

Variable(s) entered in the model: Age (continuous); LOS (continuous); MV (No/Yes); BIPAP (No/Yes).

## 5. Discussion

The present study aimed to assess the prevalence of HAI in severe and critical COVID-19 patients. This study included 723 patients with severe-critical COVID-19 who were hospitalized at Saint Georges Hospital between September 2020 and February 2021.

Our study showed 53 patients (7.3%) that developed HAI. This result is coincident with another study which showed that 8.7% of COVID-19 patients developed proven microbiologically secondary infections (10). Previous research by Iacovelli et al. reported a higher rate of HAI in their newly established COVID-19 respiratory sub-intensive care unit in Rome (11). A meta-analysis and systematic review, specifically examining bacterial co-infections and HAI in COVID-19 patients who were hospitalized, included 24 studies. The findings revealed that co-infection occurred in 3.5% of patients, while HAI were observed in 14.3% of patients (12). Numerous factors, such as varied sample sizes, population types, hospital facilities, infection prevention measures, and management levels, could contribute to the high variability of HAI across several studies. Furthermore, this high prevalence of HAI indicates the relevance and importance of addressing in it severe COVID-19 cases.

VAP (41.26%), CAUTI (28.6%), HAP (17.44%), CRBSI (6.35%), and BSI (6.35%) were the five types of HAI that were monitored. The occurrence rates of microorganisms differed across various studies. Our study showed that Gram-negative bacteria were the most common among all HAI types whereas the common two pathogens were *A. baumannii* MDR and *Enterobacterales* ESBL. In the retrospective study done by Bahceci et al., *A. baumannii* was the most prevalent in respiratory tract cultures representing 33.3% while *S. aureus* and *K. pneumoniae* each comprised 9.5% of the samples (10). In a prospective observational study, Falcone et al. reported a wide range of pathogens. *Enterobacterales* were the most commonly found microorganisms, accounting for 44.9% of the isolates. They were followed by non-fermenting Gram-negative bacilli at 15.6%, Gram-positive bacteria at 15.6%, and fungi at 5.5% (13). Furthermore, Patel et al. presented a report from Maryland, USA highlighting the rapid spread of MDR Gram-negative bacteria among COVID-19 patients. Factors contributing to the MDR spread included critical illness, high antibiotic usage, double occupancy of single rooms, and altered infection prevention protocols (14).

On the other hand, many studies compared the incidence of MDRO between the pre-COVID-19 and during the COVID-19 periods. Cogliati Dezza et al. revealed that the incidence of multi-drug resistant Gram-negative bloodstream infection was lower in patients with COVID-19 compared to those without COVID-19. In addition, pre-COVID-19 group were more likely to present *Klebiella pneumoniae* BSIs, while the COVID-19 group showed more *A. baumannii* BSIs with higher per pathogen incidence (15). In contrast, other studies reported a notable rise in infections caused by MDRO during the COVID-19 period (16, 17). Furthermore, researchers conducted a recent systematic review and meta-analysis to examine the influence of the COVID-19 pandemic on the prevalence of MDR organisms in different hospitals. They discovered a slight increase in the presence of MDR Gram-negative bacteria and a small rise in infections caused by *A. baumannii*, with no significant changes in the occurrence of Gram-positive bacteria such as MRSA or VRE infections (18). Concerning ICU patients, Ong et al. showed that COVID-19 patients had a greater rate of HAI than non-COVID-19 patients in the ICU. Critically ill COVID-19 patients may also be more vulnerable to HAI as a result of lymphopenia and compromised immune systems, in addition to having intrusive equipment. Therefore, a higher HAI rate in COVID-19 patients compared to non-COVID-19 patients may have been caused by a prolonged duration of stay, usage of invasive equipment, and decreased immune functioning (19). In addition, it is important for healthcare facilities to implement stringent infection prevention and control measures and promote appropriate antibiotic stewardship to address the increased risk of HAI caused by MDR *A. baumannii* among COVID-19 patients.

HAI and patients' sex were not significantly associated (*p* = 0.08). These findings coincide with another study (11). However, there was a significant association between HAI and patients' age; our study showed that for each 1-year increase in age, there is an increase in the risk of HAI by 1.029 times. Therefore, age is considered a risk factor for developing HAI, with a consequent greater mortality rate compared to younger patients. These findings were consistent with other studies (6–11).

The analysis of the present research data supported that DM, HTN, CAD, and CKD were statistically linked to HAI; whereas other study analysis revealed that patients with HAI also had a higher prevalence of comorbidities such as malignancy, CHF as well as atrial fibrillation (11).

Concerning COVID-19 treatment, studies showed that immunosuppression is a critical aspect in treating COVID-19 patients, as the majority of complications associated with the disease are linked to the inflammatory response and excessive release of cytokines (20). However, the administration of immunosuppressant drugs in COVID-19 patients may heighten their susceptibility to HAI; our study showed that patients with HAI were more treated with baricitinib (Janus kinase inhibitor). This result is coincident with Falcone et al.'s study where the findings suggested that patients receiving immunosuppressants (like interleukin-6 inhibitors or Janus kinase inhibitors) during their hospital stay may face an increased risk of acquiring bacterial and fungal infections (13). Also, Langford et al.'s systematic review indirectly supported these observations by providing a broader understanding of antimicrobial resistance in COVID-19 patients. They found that patients receiving IL-6 inhibitors had a higher chance to have resistant microorganisms (18). Therefore, healthcare professionals should weigh the potential benefits and risks when prescribing immunosuppressant drugs for COVID-19 patients, ensuring close monitoring and appropriate infection prevention measures.

### 5.1. Limitations

There are a few shortcomings in this research. Firstly, infected cases included are only documented by positive culture and some cases may be missing. Secondly, this study was done in a single hospital in Lebanon, with its specific epidemiology of resistant microorganisms, which makes the findings difficult to be generalized. Thirdly, the investigation whether the duration of steroids treatment and not only receiving steroids differed between patients with and without infection, since all admitted patients were on steroid during their hospital stay.

### 5.2. Strength

This is the first study in Lebanon and Middle East concerning HAI in severe-critical COVID-19 patients.

## 6. Conclusion

In conclusion, this study showed that the prevalence of HAI among severe and critical COVID-19 patients is high (7.3%). There were five types of HAI: VAP, CAUTI, HAP, CRBSI, and BSI. The most common microorganisms were mainly Gram-negative bacteria with MDR and ESBL microorganisms. HAI among severe and critical COVID-19 patients are affected by four risk factors: age, LOS, BIPAP usage, and MV. Therefore, to reduce the incidence of HAI in COVID-19 isolation wards, it is helpful to be aware of the risk factors for HAI in patients with the disease and to improve the monitoring of different susceptibility variables.

## Data availability statement

The raw data supporting the conclusions of this article will be made available by the authors, without undue reservation.

## Ethics statement

The studies involving human participants were reviewed and approved by Al-Rassoul Hospital, Beirut Lebanon. Written informed consent for participation was not required for this study in accordance with the national legislation and the institutional requirements.

## Author contributions

SF conceived and designed the study, collected the data, performed the analysis, and wrote the paper. MF contributed to the design of the article and the interpretation of data for the article. SH contributed to the analysis and the interpretation of data for the article. AA contributed to the analysis and the interpretation of data for the article, drafted the article, revised it, and approved the version to be published. All authors contributed to the article and approved the submitted version.
